# Sex Differences in Associations of Depressive Symptoms with Cardiovascular Risk Factors and Metabolic Syndrome among African Americans

**DOI:** 10.1155/2013/979185

**Published:** 2013-09-16

**Authors:** Denise C. Cooper, Ranak B. Trivedi, Karin M. Nelson, Gayle E. Reiber, Alan B. Zonderman, Michele K. Evans, Shari R. Waldstein

**Affiliations:** ^1^Northwest HSR&D Center of Excellence, VA Puget Sound Health Care System, 1100 Olive Way, Suite 1400, Seattle, WA 98101, USA; ^2^Department of Health Services, University of Washington, Seattle, WA 98195, USA; ^3^Department of Medicine, University of Washington, Seattle, WA 98195, USA; ^4^Health Services and Epidemiology, University of Washington, Seattle, WA 98195, USA; ^5^National Institute on Aging, National Institutes of Health, Baltimore, MD 21224, USA; ^6^Department of Psychology, University of Maryland Baltimore County, Baltimore, MD 21250, USA; ^7^Geriatric Research Education and Clinical Center, Baltimore VA Medical Center, Baltimore, MD 21201, USA

## Abstract

Young to middle-aged women usually have notably lower rates of cardiovascular disease (CVD) than their male counterparts, but African American women lack this advantage. Their elevated CVD may be influenced by sex differences in associations between depressed mood and CVD risk factors. This cross-sectional study examined whether relations between scores on the Center for Epidemiologic Studies-Depression (CES-D) scale and a spectrum of CVD risk factors varied by sex among African Americans (*n* = 1076; ages 30–64) from the Healthy Aging in Neighborhoods of Diversity across the Life Span (HANDLS) study. Sex-stratified multiple regressions and logistic regressions were conducted. Among women, CES-D scores correlated positively with systolic blood pressure and waist-to-hip ratio (*P*'s < .05), but inversely with high-density lipoprotein cholesterol (HDL-C) (*P* < .01). Women had twice the odds for metabolic syndrome if CES-D scores ≥16 and had a ≥14% increase in odds of hypertension, abdominal obesity, and low HDL-C with each 5-unit increase in CES-D scores. Among men, CES-D scores correlated positively with high-sensitivity C-reactive protein (*P* < .05), and odds of hypertension increased by 21% with each 5-unit increase in CES-D scores. Depressive symptoms may promote premature CVD risk in African Americans, at least in part, via CVD risk factors and prevalent metabolic syndrome, particularly in African American women.

## 1. Introduction

Young to middle-aged African American women have elevated rates of premature cardiovascular morbidity and mortality [1, 2], including greater prevalence of cardiovascular disease (CVD) [3] and higher cumulative incidence of heart failure before age 50 [4] than African American men. These sex differences in CVD before age 65 have not been well studied in African Americans. Given that clinical and subclinical levels of depressive symptoms have been linked to CVD [5], the elevated levels of depressed mood observed in African American women [6–8] might contribute to their increased risk for CVD.

Depressed mood has been linked to CVD risk factors and to metabolic syndrome [7–37], with such relations often differing according to sex [15–18, 20, 25–28, 31–37]. While research on these sex differences is largely based on predominantly white samples in the USA [7, 8, 16–20] and on samples in other countries [14, 15, 21–33], several investigations in the USA have examined racially diverse samples that included substantial representation by African Americans [8, 34–38]. However, this literature is limited by its dearth of research specifically investigating sex differences in African Americans, with the exception of two studies that found that depressive symptoms were not associated with blood pressure [8] or high-sensitivity C-reactive protein (hsCRP) [38] in either African American women or African American men. 

Accordingly, this study aims to addresses gaps in the literature by cross-sectionally examining how depressive symptoms relate to select CVD risk factors—blood pressure, fasting lipid and glucose levels, waist-hip ratio (WHR), and hsCRP—in young-to middle-aged African American women and men. Both continuous outcome measures (e.g., total cholesterol) and traditional clinical cut-offs (e.g., hypercholesterolemia) were examined. Prevalent metabolic syndrome also was assessed.

## 2. Methods

### 2.1. Participants

The sample includes 1,076 African American men and women, ages 30 to 64 years, who participated in the first wave (i.e., baseline assessment) of the larger Healthy Aging in Neighborhoods of Diversity across the Life Span (HANDLS) study. HANDLS is an ongoing 20-year epidemiological study of health disparities in Baltimore, Maryland initiated in 2004 by the National Institute on Aging Intramural Research Program at the National Institutes of Health. As reported previously [39], its baseline is representative of working age adults recruited as a fixed cohort from an area probability sampling of thirteen Baltimore City neighborhoods (contiguous census tracts) between 2004 and 2008. Excluded were pregnant women, individuals with AIDS, and cancer patients treated with chemotherapy, radiation, or biological treatments within past 6 months. Of the 3720 individuals enrolled during the baseline assessment, 2198 were African American. Of these African American participants, 1600 had completed a physical examination. For the purposes of this study, we limited our data to the 1076 African American participants for whom we had complete data on the primary study variables along with the medication and lifestyle covariates. The study received Institutional Review Board approval. Participants provided written informed consent.

### 2.2. Procedures

The HANDLS study procedures have been described in detail previously [39]. To summarize aspects of the HANDLS protocol relevant to the current analyses, researchers collected demographic data and other background information in an interview at the participant's home. During a standard all-day appointment on a mobile research vehicle parked in the participant's community, medical staff obtained a fasting blood sample, blood pressure, and anthropometric measurements, followed by a physical examination and medical history, collection of data on medication usage, and administration of the Center for Epidemiologic Studies-Depression (CES-D) scale.

### 2.3. Measures

#### 2.3.1. Predictor Variable: Depressive Symptoms

CES-D: the 20-item CES-D scale assesses the frequency of depressive symptoms experienced in the past week on a 4-point Likert scale ranging from “0” (“rarely or none of the time (less than one day”) to “3” (“most or all of the time (5 to 7 days”) [40]. Scores range from 0 to 60. CES-D scores ≥16 suggest clinically significant levels of depression [40].

#### 2.3.2. Outcome Variables: Cardiovascular Risk Factors and Metabolic Syndrome

Blood pressure/hypertension: systolic and diastolic blood pressure rates (SBP and DBP) were measured twice using the brachial artery auscultation method after seated participants rested with legs uncrossed for 5 minutes. The two readings (one reading from each arm) were averaged for the analyses. Hypertension was defined as average SBP ≥140 mmHg or DBP ≥90 mmHg or prior diagnosis of hypertension (per self-reported medical history) or use of antihypertensive medication (per participants' pharmacy bottles) [1].

*Waist-Hip Ratio/Abdominal Obesity*. Waist-hip ratio (WHR) was calculated as the waist circumference divided by the hip circumference. Waist circumference was measured with a tape measure placed at the midpoint between the lower rib margin and iliac crest. Hip circumference was measured at the widest point of hips and buttocks. Abdominal obesity was defined as WHR ≥0.90 in men and ≥0.85 in women [41].

*Lipids/Dyslipidemia, Glucose/Diabetes, and hsCRP/High Inflammation.* After an overnight fast, blood samples were obtained and standard laboratory methods (Quest Diagnostics: Chantilly, VA) were used to assess total serum cholesterol (CHOL), low- and high-density lipoprotein cholesterol (LDL-C, HDL-C), triglycerides (TG), glucose (GLU), and hsCRP. Dyslipidemia subtypes were defined as high CHOL (>240 mg/dL), low HDL-C (<40 mg/dL in men or <50 mg/dL in women), high LDL-C (>160 mg/dL), or high TG (>200 mg/dL) [1]. Diabetes was defined as high GLU (≥126 mg/dL) and/or prior diagnosis of diabetes (per self-reported medical history) and/or use of antidiabetic medication (per participants' pharmacy bottles) [1]. High inflammation was defined as high hsCRP ≥3 mg/L [42].

*Metabolic Syndrome.* The criteria recommended by the National Cholesterol Education Program (NCEP) Adult Treatment Panel III [43] were used to identify metabolic syndrome. It was defined as ≥3 of the following components: waist circumference >102 cm in men or >88 cm in women; blood pressure ≥130/85 mmHg; fasting GLU ≥110 mg/dL; TG ≥150 mg/dL; and HDL-C <40 mg/dL in men or <50 mg/dL in women [43].

#### 2.3.3. Covariate Measures: Sociodemographic, Lifestyle, and Biomedical Variables

Sociodemographic: data were collected on age, sex, and poverty status during the home interview. Poverty was defined as family income <125% of the Federal poverty threshold [39]. 

Lifestyle: self-reported medical histories were the data source for covariates of lifetime smoking history (i.e., have ≥100 cigarettes been smoked in lifetime?), lifetime drug history (i.e., have cocaine, opiates, and/or cannabis been used ≥5 times in lifetime?), and past week use of alcohol (i.e., has alcohol been consumed in past 7 days?). 

Biomedical: BMI was calculated as weight in kilograms divided by height in meters squared (kg/m^2^). Self reported medical histories were the source of data on prior diagnoses of hypertension/CVD, diabetes, and menopause status (i.e., surgical removal of both ovaries and/or at least 6 months without a menstrual period in women ≥50 years of age). Staff recorded prescriptions, which were subsequently aggregated into antihypertensive, antidiabetes, antidepressant, cholesterol-reduction, and hormone replacement therapy medications based on self-reported use or based on pharmacy bottles participants brought to their examinations. 

### 2.4. Data Analytic Procedures

Descriptive analyses and *t*-tests were conducted to characterize the sample and how men and women differed with regard to the means of study variables. To examine how depressive symptoms may differentially relate to CVD risk factors among African American women and among African American men, sex-stratified analyses were conducted in the following two ways. Due to the clinical cut-offs for CVD risk factors having an uncertain generalizability to African Americans, we examined CVD risk factors both as continuous scores (in multiple regressions) and as clinical cut-offs (in logistic regressions). 

For multiple regression models, the outcome variables were SBP, DBP, WHR, CHOL, HDL-C, LDL-C, TG, GLU, and hsCRP, whereas the outcomes in logistic regression models were hypertension, high WHR, hypercholesterolemia, low HDL-C, high LDL-C, high TG, diabetes, and high hsCRP. In both sets of analyses, a standard set of covariates was entered in the first step/block, and CES-D scores (continuous) were entered as the primary predictor variable in the last step/block. (To enhance clinical interpretability of each odds ratio (OR), we divided the original CES-D scores by 5 and used these modified continuous CES-D scores in the logistic regressions; thus, the ORs shown for CVD risk factors are for every 5-point increase in CES-D scores.) The covariates were age, poverty status, and BMI; histories of CVD, hypertension, and diabetes; medications (antihypertensive, antidiabetes, cholesterol reduction, and antidepressant); hormone replacement therapy and menopause status (women only); and history of smoking, drug use, and recent alcohol use. Selection of covariates was based on prior literature [8, 11, 12, 24–26, 44] or significant correlations (*P* < .05) with the outcome variables. Covariates were omitted from certain models when the outcome variable was similar to the covariate (e.g., BMI was omitted as a covariate in the WHR/abdominal obesity models and history of hypertension was omitted as a covariate in the SBP/DBP/hypertension models).

Secondary analyses utilized logistic regression to examine whether clinically significant depression symptoms (i.e., CES-D scores ≥16) were associated with increased odds of meeting criteria for metabolic syndrome [43]. Logistic regressions were adjusted for the same covariates that were used in the primary analyses.

While the overall sample included 1,076 adults, data on fasting CHOL, HDL-C, LDL-C, TG, GLU, and hsCRP were available only for a subset (*n*'s ranged from 491 to 592). Consequently, sample sizes were reduced in all analyses involving these measures, as well as metabolic syndrome. 

## 3. Results

The mean age was 48 years old, and about 43% of participants had household incomes below 125% of the poverty level for their family size (Table 1). Women had higher mean CES-D scores (*P* < .05), a greater proportion with CES-D scores ≥16, and a greater percentage who used antidepressant medications (*P's* < .01) than men. Although men had higher mean DBP levels than women (*P* < .05), greater proportions of women had hypertension (based on current blood pressure, prior diagnosis, or antihypertensive use) and a medical history of CVD (*P*'s <.001). In addition, women met more clinical cut-offs for low HDL-C (*P* < .001), elevated hsCRP (*P* < .001), and high WHR (*P* < .01) than men. A greater proportion of men than women had high TG (*P* < .05) and alcohol consumption within the last week (*P* < .001), as well as lifetime smoking of ≥100 cigarettes and lifetime drug history of using cocaine, opiates, or marijuana ≥5 times (*P's* < .001).

 Depressive symptoms were differentially associated with CVD risk factors (continuous scores) according to sex (Table 2). Among women, greater CES-D scores were significantly associated with higher levels of SBP and WHR (*P*'s <.05) and lower levels of HDL-C (*P* < .01) in regressions, after adjustment for covariates. The associations of CES-D scores with WHR and HDL-C, but not SBP, were indicated in unadjusted regressions (*P*'s <.05) among women. In contrast, men showed only a significant association between greater CES-D scores and higher levels of hsCRP (*P* < .05) in the adjusted regression model, as well as the unadjusted regression model.

 Adjusted logistic regression models of CES-D scores on clinical cut-offs for CVD risk factors also revealed sex differences (Table 3). Among women, increasing CES-D scores were significantly associated with increased odds of having hypertension (adjusted OR = 1.16; 95% confidence interval (CI): 1.02, 1.31), high WHR (adjusted OR = 1.14; 95% CI: 1.001, 1.29), and low HDL-C (adjusted OR = 1.22; 95% CI: 1.03, 1.45). Thus, for every 5-point increase on the CES-D, the odds of hypertension, high WHR, and low HDL-C increased in women by 16%, 14%, and 22%, respectively. Like their female counterparts, men showed a significant association between higher CES-D scores and the adjusted odds of having hypertension (adjusted OR = 1.21; 95% CI: 1.03, 1.43), with the adjusted odds of hypertension in men increasing by 21% for every 5-point increase on the CES-D. While CES-D scores were significantly associated with hypertension in men in the unadjusted logistic regression, they showed only marginal or nonsignificant associations with hypertension, high WHR, and low HDL-C before adjustments for covariates and a significant association with high TG that became nonsignificant after entry of hormone replacement therapy and the other covariates in the adjusted logistic regression model.

Metabolic syndrome in depressed versus nondepressed (i.e., CES-D scores ≥16 versus <16) participants varied by sex (Table 4). Though the unadjusted model indicated a nonsignificant relationship, the model adjusted for covariates indicated that women with CES-D scores ≥16 had greater odds of metabolic syndrome, with adjusted OR of 2.64 (95% CI: 1.19, 5.83). Men did not show a significant association between depression status and metabolic syndrome in adjusted or unadjusted models. 

## 4. Discussion

 Among this community-based samples of young to middle-aged African Americans, higher levels of depressive symptoms in women were related to greater SBP and odds of hypertension, greater WHR and odds of abdominal obesity, lower HDL-C with greater odds of low HDL-C, and greater odds of metabolic syndrome. Depressive symptoms in men were associated with greater odds of hypertension and with higher levels of hsCRP. Despite only slightly higher mean scores on the CES-D in women, the associations between depressive symptoms and CVD risk factors differed notably by sex. Although very few prior studies have explicitly focused on African American samples, there is a relatively large literature available on sex differences in the association of depressive symptoms with cardiovascular risk factors. Comparison with prior literature is discussed below.

### 4.1. Depressive Symptoms, Blood Pressure Levels, and Hypertension

Our findings that depressive symptoms were positively associated with SBP levels among women were consistent with findings from an all-female study of relatively healthy African American girls and women [45] that adjusted for antihypertensive medications and other confounders and with a study of healthy, medication-free adults (ages 30–70) in Germany [26]. In contrast, most of the prior sex differences literature [8, 16, 18, 38], including two studies of African American women and men [8, 38], reported no associations between depressive symptoms and SBP or DBP in either sex. In general, those prior studies with consistent findings [26, 45] accounted for medications and other confounders similar to those accounted for in the current study whereas prior studies with inconsistent findings did not [8, 16, 18, 38].

Results of the present study further indicate an association between higher CES-D scores and greater odds of hypertension in both African American men and women. Although no directly comparable studies of African American men were found, a smaller all-female study of relatively healthy African American girls and women (*n* = 159; ages 13 to 90) noted an association between CES-D scores ≥16 and hypertension [45]. Furthermore, partially consistent findings were noted in a study of racially diverse young adults [34] in which women showed a positive association between lifetime history of major depression and a measure of high blood pressure with a lower threshold (i.e., SBP/DBP ≥130/85 mmHg) and scope (i.e., prior diagnosis of hypertension was not included) than hypertension as defined in the current study. Lastly, a prospective study reported that CES-D scores were linked to incidence of hypertension (after controlling for a variety of confounders) in African American adults [9]. 

 Thus, although similar depression-hypertension findings have been noted in select studies of African Americans that did not examine sex differences [9, 45], our results are inconsistent with several prior studies of predominantly White or Asian samples, which showed no associations in either sex [28–30, 33]. However, outside of the sex differences literature on depression and hypertension, which is primarily cross-sectional, the larger literature mirrors the current findings in providing support for a link between depression and hypertension. A meta-analysis of prospective studies found that depression was associated with a 42% increase in the risk of hypertension incidence [46]. 

### 4.2. Depressive Symptoms, WHR Levels, and Abdominal Obesity

Depressive symptoms were associated with a greater WHR and higher odds of abdominal obesity in women, but not men. No directly comparable studies of African American women were located. However, a smaller study of African American adults (*n* = 253; (ages 18 to 60 years old) reported a positive association between CES-D scores and WHR levels after controlling for socioeconomic confounders [10]. The more general literature on sex differences in the relations between depressed mood and WHR [14, 15, 22–24] ranges from studies reporting unadjusted or modestly adjusted positive results in both men and women to studies reporting no association in either sex after accounting for confounders similar to the current study. However, the latter studies finding no associations in either sex were conducted in the Netherlands [24] and China [14] with samples of older adults.

### 4.3. Depressive Symptoms, Lipid Levels, and Dyslipidemias

We found that higher CES-D scores were related to lower HDL-C levels and to the clinical cut-off for low HDL-C in women only. This is inconsistent with prior literature reporting no association in either sex [16, 27, 28, 30, 31, 33, 34, 36], including a study reporting data for African American women and men [38]. However, studies of predominantly white samples in the USA [18] and in Europe [25, 26, 29] have indeed reported associations of depressive symptoms and HDL-C among women [25, 26] after control of confounders similar to the current study, while other studies with less control of confounders report associations in both women and men [18, 29]. In general, these studies differed from the present study by assessing more severe depression using cut-offs for clinical depression [18, 25, 26] or history of major depression [29] instead of continuous depressive symptom scores. Thus, it is unknown whether similar associations between depression and HDL-C in predominately white samples [18, 25, 26, 29] would be found if continuous depressive symptom scores were used to capture the continuum of mild to severe levels of depression. Overall, our finding that depressive symptoms were associated with HDL-C only in women is consistent with the larger literature, as shown by a meta-analytic review that reported a significant association in women only [47].

### 4.4. Depressive Symptoms, hsCRP Levels, and High Inflammation

The positive association between CES-D scores and hsCRP levels among African American men (not women) noted here is inconsistent with several reports of no association in either sex [19, 20], including a study of young to middle-aged African Americans [38]. The inconsistency between the latter study of African Americans [38] and the current study may be partly due to our use of a different depressive symptom scale and our adjustment for different confounders (e.g., medications, history of CVD, and drug history). However, a study in Brazil found positive relations between CES-D scores and C-reactive protein (not hsCRP; assay was not high sensitivity) among men, but an inverse association among women [32]. 

 In contrast to continuous hsCRP, high levels of hsCRP (defined as hsCRP ≥3 mg/L) were not associated with CES-D in men, possibly due to statistical power. Prior studies with a racially diverse sample found a link between history of major depression and C-reactive protein levels ≥.22 mg/dL [12, 35], but this work did not examine hsCRP, as the assay used was not high sensitivity. Overall, the positive association observed in the current study between continuous CRP levels and depressive symptoms in African American men is consistent with the findings of a meta-analysis, which reported that CRP and depression were significantly related in men, but not women [48].

### 4.5. Depressive Symptoms and Metabolic Syndrome

The more than doubling of odds for metabolic syndrome among women with clinically relevant levels of depressive symptoms in this study is notable, as metabolic syndrome is associated with a 2-fold increase in the risk of myocardial infarction, stroke, CVD incidence, and CVD mortality [49]. A meta-analytic review of the overall literature similarly found that depression was significantly related to metabolic syndrome and that the association was bidirectional [50]. However, the literature offered no comparable research that examined African Americans for sex differences in the relationships. A study of older White and African American adults (70–79 years of age) reported an association between depressive symptoms and metabolic syndrome only in Whites, not African Americans, but did not account for antidepressant use [51]. Only a study of older adults (≥75 years old) in Italy was consistent with the current study both with regard to findings and with accounting for similar confounders, including antidepressants, alcohol use, menopausal status/hormone replacement therapy (indirectly via age exclusion of <75 years old), and drug use (indirectly limited drug use confounding via sample age) [28]. Additional studies reported similarly positive results among young women [34] and young/middle-aged/older women [18, 33] but accounted for few, if any, of the aforementioned confounders, which limits interpretation and generalization of findings across these samples. Our findings were inconsistent with similar studies that found an association between depression and metabolic syndrome among men only [27] or among neither men nor women [26, 29, 30]. 

### 4.6. Integration and Underlying Mechanisms

 It is clear that prior research on the associations between depressive symptoms and cardiovascular risk factors has yielded a highly mixed and methodologically dissimilar body of work. This relates to differential methods of sampling, operationalizing depression, risk factor outcomes, and variable adjustment for confounders. Of perhaps the greatest concern is the need for further research that accounts for critical medical confounders and medications, as well as medication adherence [44] to determine whether other samples of African Americans and of women and men from other racial groups show similar relations between current depressive symptoms and cardiovascular risk factors as noted here.

 An additional consideration is that the geographic location of any particular study also may influence its findings. Most of the literature on sex differences in the association between depression and CVD risk factors is derived from samples outside of the USA [15, 21–33]. The participants in the HANDLS study reside in Baltimore, MD, a city that is 63% African Americans and 32% White. Baltimore has a wide range of socioeconomic status, but a moderately high percentage of residents at or below the poverty line [39]. Relative to living in other countries or in other cities/regions of the USA, living in Baltimore may confer both similar and differential mechanisms that could link depressive symptoms to CVD risk factors. These mechanisms may further differ by sex. Examples include various lifestyle factors such as dietary habits and engagement in physical activity (that may be limited by barriers unique to select neighborhoods), smoking, alcohol consumption, and illicit drug use. In that regard, despite our statistical adjustment for the latter three variables, the likelihood of residual confounding remains, and a more detailed assessment of quantity, frequency, and duration of use will be needed. Other potentially relevant factors to this population are limited access to healthcare and cultural mistrust of the healthcare system.

 Lastly, stress-related autonomic nervous system, neuroendocrine, and immune pathways may be relevant and may vary by sex. For example, research on predominantly White samples suggests that autonomic nervous system activity is influenced more by the parasympathetic system among women, while the sympathetic system is more influential among men [52, 53]. As individuals and as residents of certain neighborhoods, African Americans, particularly those with lower socioeconomic status, are often exposed to multiple chronic psychosocial and environmental stressors (e.g., racial discrimination, crowding, crime, and financial strain) [54]. Recent work suggests that social class may be differentially associated with dysregulation of biological systems among African Americans according to sex, such that lower socioeconomic status is associated with dysregulation of metabolic and immune function in African American women and with dysregulation of neuroendocrine functions in African American men [54]. 

### 4.7. Strengths and Limitations of the Study

This study contributes to the literature on depression and CVD risk factors by expanding the information available on sex differences in general, but particularly within African Americans. The study is strengthened by its examination of a spectrum of risk factors using both continuous measures and established clinical cut-offs. It is also strengthened by its adjustments for confounders that are often overlooked in similar studies (e.g., medications, CVD, menopause status, and drug use) and by its utilization of the CES-D scale, which has been widely used in cardiovascular research and allows for comparisons across studies. Further, the use of continuous CES-D scores provided evaluation of the associations with CVD risk factors across the spectrum of depressive mood severity.

The study is limited by its use of self-reported measures (e.g., CES-D and lifestyle data), dichotomized variables (e.g., poverty, smoking, and drug use), and an alcohol measure that assessed how recently alcohol was consumed rather than quantity. It also has limitations in its generalizability to other research settings and other populations. Approximately 43% of this sample was impoverished (per Federal guidelines). While this level of poverty was representative of the Baltimore City neighborhoods that were sampled, the results may have limited generalizability to African American populations in other locations that have fewer people living in poverty. Men had lower mean CES-D scores and a lower proportion with CES-D ≥16 than women. While it is possible that this may have influenced our results, we do not feel that the lower level of depressive symptoms among men interfered with our ability to detect associations in this group because the range of continuous CES-D scores in men was not truncated and the proportion of men with CES-D ≥16 was nearly 22%. Finally, its cross-sectional design cannot establish temporal associations between depression and CVD risk factors.

## 5. Conclusions

Overall, our findings suggest that young to middle-aged African American women and men living in Baltimore, MD, show similar relations between depressive symptoms and hypertension. Although depressive symptoms were also related to greater hsCRP in AA men, it is particularly concerning that depressed mood in African American women was further linked to increased odds of abdominal obesity, low HDL-C, and metabolic syndrome. It is thus possible that depressed mood may, in part, be a contributing factor to why young-to-middle aged African American women lack the same degree of female advantage in cardiovascular health found among similarly aged women of other racial/ethnic groups compared to their male counterparts. There is a dire need for better efforts in screening, prevention, and intervention with cardiovascular risk factors among African Americans, particularly women. Further research is needed to assess the mechanisms that underlie the differential associations of depressive symptoms with cardiovascular risk profiles in African American men and women. 

## Figures and Tables

**Table 1:** Sample characteristics and sex differences.

Variable (raw scores)	Total (*n* = 1076) Mean (SD) or %	Women (*n* = 582) Mean (SD) or %	Men (*n* = 494) Mean (SD) or %
Sociodemographic and lifestyle covariates			
Age (yrs)	48.2 (9.1)	48.3 (9.3)	48.0 (8.9)
Poverty (per Federal guidelines) %	42.5	43.8	41.1
Smoking history (lifetime ≥100 cigarettes)%***	71.4	65.0	78.8
Alcohol (consumed in past week)%***	41.4	34.4	49.5
Drug History (lifetime coke/opiate/cannabis ≥5x)%***	58.7	45.7	73.7
Biomedical covariates			
BMI (kg/m^2^)***	30.1 (7.8)	32.3 (8.6)	27.4 (5.8)
Medical History of CVD %***	22.9	27.6	17.3
Medical history of hypertension***	44.9	51.2	37.7
Medical history of diabetes %	17.4	17.9	16.7
Antihypertensive medication use %***	35.6	41.7	28.5
Antidiabetes medication use %	13.4	13.7	13.0
Cholesterol-reduction medication use	12.6	14.1	11.0
Antidepressant medication use %**	9.4	12.0	6.5
Hormone replacement therapy use %	0.7	1.2	n/a
Menopause %	23.3	43.4	n/a
Predictor and outcome variables			
CES-D*	11.3 (7.8)	11.8 (8.5)	10.7 (6.8)
CESD ≥ 16**	25.8	29.5	21.6
Hypertension^a^ %***	52.6	57.8	46.6
Mean SBP (mmHg)	122.2 (17.4)	122.8 (18.5)	121.4 (16.1)
Mean DBP (mmHg)*	73.5 (11.2)	72.7 (11.0)	74.5 (11.4)
WHR obesity: ≥0.90 ♂ and ≥0.85 ♀ %**	67.2	71.4	62.3
Mean WHR***	0.92 (0.01)	0.90 (0.1)	0.94 (0.1)
Mean waist circumference (cm)***	99.0 (17.5)	102.1 (18.3)	95.4 (15.8)
Hypercholesterolemia^b^ %	20.2	22.7	17.6
Mean CHOL (mg/dL)	183.7 (44.9)	184.6 (38.8)	182.9 (50.7)
Low HDL-C: <40 ♂ and <50 ♀ (mg/dL) %***	33.7	43.1	23.6
Mean HDL-C (mg/dL)	55.3 (18.4)	55.9 (16.0)	54.6 (20.7)
High LDL-C: >160 mg/dL %	7.4	8.2	7.2
Mean LDL-C (mg/dL)	107.7 (37.1)	108.4 (34.8)	106.9 (39.4)
High TG: >200 mg/dL %	6.1	4.3	8.0
Mean TG (mg/dL)	105.7 (85.4)	101.3 (50.6)	110.3 (111.1)
Diabetes^c^ %	18.7	20.8	16.3
Mean GLU (mg/dL)	106.5 (46.3)	110.0 (53.1)	102.8 (37.4)
High hsCRP: ≥3 mg/L %***	38.9	46.9	30.4
Mean hsCRP (mg/L)	5.9 (14.6)	6.7 (12.5)	5.0 (16.5)

^a^Hypertension: SBP ≥ 140 or DBP ≥ 90 or prior diagnosis or use of antihypertensive medication.

^b^Hypercholesterolemia: fasting CHOL > 240 mg/dL or use of cholesterol-reduction medication. ^c^Diabetes: fasting GLU: ≥126 mg/dL or prior diagnosis or use of antidiabetes medication.

Sex differences: ***P < .001; **P < .01; *P < .05.

**Table 2:** Regression coefficients of associations between CES-D scores and CVD risk factors (continuous scores) by sex.

Outcome variable	Women				Men			
	CES-D scores (0 to 60 points)				CES-D scores (0 to 60 points)			
	*N*	*β*	*B* (SE)	*P*	*N*	*β*	*B* (SE)	*P*
SBP (mmHg)	573	.08	.001 (.00)	.042*	494	.06	.000 (.00)	.183
DBP (mmHg)	573	.08	.001 (.00)	.077^‡^	494	−.01	.000 (.00)	.861
WHR	582	.10	.001 (.00)	.016*	483	−.04	.000 (.00)	.346
CHOL (mg/dL)	254	−.10	−.469 (.29)	.111	239	.05	.378 (.53)	.480
HDL-C (mg/dL)	254	−.18	−.355 (.12)	.003**	238	.07	.216 (.19)	.257
LDL-C (mg/dL)	254	−.03	−.137 (.26)	.598	237	.07	.391 (.41)	.338
TG (mg/dL)	254	.02	.117 (.40)	.767	239	−.05	−.771 (1.2)	.504
GLU (mg/dL)	308	.01	.078 (.28)	.782	284	−.08	−.561 (.35)	.114
hsCRP (mg/L)	254	.00	.001 (.10)	.988	238	.17	.421 (.17)	.011*

Significance values for the CES-D in multiple regression models: **P < .01; *P < .05; ^‡^ *P* = .07.

Data shown are for multiple regressions after adjustments for age, poverty status, BMI; histories of CVD, hypertension, and/or diabetes; medication use (i.e., antihypertensive, antidiabetes, cholesterol reduction, antidepressants, and hormone replacement therapy); menopausal status; lifetime histories of smoking and drug use; and alcohol use in past week. (Exceptions: covariates BMI, history of hypertension, and history of diabetes were omitted from the models for WHR, SBP/DBP, and fasting glucose, resp. Menopause status and hormone replacement therapy were omitted from models for men.)

**Table 3:** Adjusted odds of associations between the CES-D (continuous scores) and CVD risk factors (clinical cut-offs) by sex.

Outcome variable	Women				Men			
	CES-D scores (0 to 60 points)				CES-D scores (0 to 60 points)			
	*N*	Odds ratio	95% confidence interval	*P*	*N*	Odds ratio	95% confidence interval	*P*
Hypertension								
High SBP ≥140 or DBP ≥90 mmHg (or prior hypertension diagnosis/treatment)	573	1.16	1.02, 1.31	.022*	494	1.21	1.03, 1.43	.022*
Abdominal obesity								
High WHR ≥0.9 ♂ and ≥0.85 ♀	562	1.14^a^	1.00, 1.29	.048*	483	0.99	0.85, 1.15	.869
Dyslipidemia								
Hypercholesterolemia: fasting CHOL >240 mg/dL (or treatment)	268	0.98	0.79, 1.22	.876	247	0.94	0.71, 1.24	.652
Low HDL-C <40 ♂ and <50 ♀ mg/dL	254	1.22	1.03, 1.45	.019*	238	0.91	0.70, 1.16	.438
High LDL-C: >160 mg/dL	254	1.09	0.81, 1.46	.584	237	1.45	0.97, 2.16	.073^‡^
High TG >200 mg/dL	254	1.17	0.81, 1.70	.407	239	1.09	0.77, 1.55	.631
Diabetes								
Fasting GLU ≥126 mg/dL or prior diabetes diagnosis/treatment	321	0.96	0.78, 1.18	.677	292	1.05	0.79, 1.40	.751
High inflammation								
High hsCRP ≥3 mg/L	254	0.98	0.81, 1.18	.845	238	1.16	0.89, 1.50	.269

Odds ratios for outcome variables are based on a 5-unit change in CES-D score.

Significance for the CES-D in models: *P < .05; ^‡^ *P* = .07. ^a^The confidence interval includes 1 due to rounding.

Data shown are for logistic regressions after adjustments for age, poverty status, and BMI; histories of CVD, hypertension, and/or diabetes; medication use (antidiabetes, antihypertensive, cholesterol reduction, anti depressants, and hormone replacement therapy); menopausal status; lifetime histories of smoking and drug use; and alcohol use in past week. (Exceptions: covariates BMI, history of hypertension/antihypertensive medications, history of diabetes/antidiabetes medication, and cholesterol-reduction medication were omitted from the models for WHR, hypertension, diabetes, and hypercholesterolemia, resp. Menopause status and hormone replacement therapy were omitted from models for men.)

**Table 4:** Adjusted odds of associations between the CES-D 16 and metabolic syndrome by sex.

Outcome variable	Women				Men			
	CES-D scores ≥16				CES-D scores ≥16			
	*N*	Odds ratio	95% confidence interval	*P*	*N*	Odds ratio	95% confidence interval	*P*
Metabolic syndrome	251	2.64	1.19, 5.83	.017	233	1.35	0.43, 4.24	.603

Odds adjusted for age, poverty status, and BMI; histories of CVD, hypertension, and/or diabetes; medication use (antidiabetes, antihypertensive, cholesterol reduction, and antidepressant); hormone replacement therapy and menopausal status (in women); lifetime histories of smoking and drug use; and alcohol use in past week.

## Abbreviations

BMI: Body mass index
CES-D: Center for Epidemiologic Studies-Depression
CI: Confidence interval
CHOL: Total serum cholesterol
CRP: C-reactive protein
hsCRP: High-sensitivity C-reactive protein
CVD: Cardiovascular disease
DBP: Diastolic blood pressure
GLU: Glucose
HANDLS: Healthy Aging in Neighborhoods of Diversity across the Life Span Study
HDL-C: High-density lipoprotein cholesterol
LDL-C: Low density lipoprotein cholesterol
OR: Odds ratio
SBP: Systolic blood pressure
TG: Triglycerides
WHR: Waist to hip ratio.
